# A Simple Model to Determine the Efficient Duration of Exams

**DOI:** 10.1177/0013164420963163

**Published:** 2020-10-16

**Authors:** Jules L. Ellis

**Affiliations:** 1 Radboud University Nijmegen, Nijmegen, the Netherlands

**Keywords:** test length, reliability, study load, efficient, measurement errors, costs

## Abstract

This study develops a theoretical model for the costs of an exam as a function of its duration. Two kind of costs are distinguished: (1) the costs of measurement errors and (2) the costs of the measurement. Both costs are expressed in time of the student. Based on a classical test theory model, enriched with assumptions on the context, the costs of the exam can be expressed as a function of various parameters, including the duration of the exam. It is shown that these costs can be minimized in time. Applied in a real example with reliability .80, the outcome is that the optimal exam time would be much shorter and would have reliability .675. The consequences of the model are investigated and discussed. One of the consequences is that optimal exam duration depends on the study load of the course, all other things being equal. It is argued that it is worthwhile to investigate empirically how much time students spend on preparing for resits. Six variants of the model are distinguished, which differ in their weights of the errors and in the way grades affect how much time students study for the resit.

## Introduction

Exams at universities typically take between 0.5 and 5 hours, but what is the optimal duration? Recently, this was discussed by the faculty of the psychology department of the Radboud University, and several faculty members suggested that the courses with high study load require longer exams. The other side of this argument is that short exams, of say 1 hour, would suffice for small courses. Since I am Chairman of the Examination Board of the psychology program, my opinion on this matter was asked. My first thought on this was that a shorter exam will have less questions, and consequently a lower reliability, and that this might breach the common standards of reliability. For example, the Dutch Committee on Tests and Testing states that the reliability of a test being used for “important decisions at individual level” should be at least 0.80 (Evers et al., 2019, p. 34). However, the department is not bound by this regulation, and Ellis (2013) argued against such arbitrary reliability standards. Therefore, let us consider explicitly what the problems of a short test would be.

The main problem of a short test is that the inevitable measurement errors will be relatively large. Consequently, there will be more students who fail the exam while they should have passed, and more students who pass the exam while they should have failed. But to how many students does this happen? In order to make an informed decision, we want to quantify this. The next section will develop a simple classical test theory model for this.

The next question is what the costs of such incorrect decisions are. If the student fails, the student will retake the exam later, and for this the student needs additional study time. The present study will consider this time as the costs for the student. If the student passes undeservedly, it is harder to quantify the costs. In this case, there are usually no extra costs for the student, but there may be costs for the institution in terms of reputation damage if future employers or teachers note the lack of skills of some students who passed this exam. However, this kind of costs is much harder to quantify, and therefore it will be ignored in most of the article.

Having a long exam, on the other hand, has also costs, because all students of the course will have to spend time on this. Based on these ingredients, the optimum exam duration may be defined as the exam duration for which the total costs in the student population are minimal, where the total costs are computed as (1) the hours extra study time for students who failed incorrectly plus (2) the hours spent by all students on the examination. This article will demonstrate that this optimum is easily computed if the relevant statistics are known from a previous administration.

Obviously, there are in fact also other costs for the student, such as emotional stress. There will also be additional costs for the institution, such as the need of a bigger exam hall during the resit if more students fail. Furthermore, the costs of correcting the exam are ignored, which might be defensible for multiple choice exams that are automatically processed. Furthermore, one might argue that an item response theory model is needed instead of classical test theory. All these improvements will be ignored for the sake of simplicity of the model.

The next section describes how the present article is related to articles with similar objectives. The following sections will explicate the test score model and explain how the probability of an incorrect fail on the exam can be derived from it, and include the two costs in the model and define the objective function. After illustrating this model with a real exam in discrete time, it will be shown that with continuous time, the objective function is convex and has a unique minimum. The following section will investigate numerically how the minimum depends on the parameters. Several other versions of the model are discussed briefly.

## Previous Optimization Approaches

This section will sketch how the method developed in the present article differs from earlier approaches. Several authors have studied how one can optimize generalizability coefficients, reliability coefficients, or validity coefficients, or how one can minimize decision error rates. In many cases this was studied in the context of a budget constraint (e.g., Allison et al., 1997; Ellis, 2013; Liu, 2003; Marcoulides, 1993, 1995, 1997; Marcoulides & Goldstein, 1990a, 1990b, 1992; Meyer et al., 2013; Sanders, 1992; Sanders et al., 1991) or fixed total testing time (e.g., Ebel, 1953; Hambleton, 1987; Horst, 1949, 1951). Alternatively, one may minimize the costs of measurement given constraints on the generalizability coefficient or error variance (e.g., Peng et al., 2012; Sanders et al., 1989), which is a similar problem. In these cases, the measurements have multiple facets (such as items and subjects) or multiple components (such as subtests), each associated with different costs or durations. The optimization achieves a balance of these facets or components. The present study, in contrast, does not assume a priori constraints on the budget, testing time, generalizability coefficient, error variance, or error rate. Instead, it specifies not only the costs of measurements but also the costs of decision errors—unlike the studies cited above. These two kinds of costs will be balanced via minimization of the expected loss.

Many of the articles cited above use the Spearman–Brown formula, and this will be used in the present study too. Several articles assume continuous parameters, because this permits the use of derivatives. The present study will do that too.

A different branch of psychometric optimization is the selection of items in Computerized Adaptive Testing (e.g., Wiberg, 2003), maximizing Cronbach’s alpha (Flebus, 1990; Thompson, 1990), or creating test forms (e.g., Raborn et al., 2020). In these studies there is a constraint on the test length or the error variance. The present study does not use such constraints. Furthermore, these studies assume that the test items have different psychometric parameters, which are being used in the optimization, while the present study assumes test items that are equivalent with respect to psychometric parameters and costs.

Assuming that errors cost something is naturally done in decision problems in economics or industry. In educational testing, such assumptions are usually avoided because there is no obvious, generally agreed-upon method to quantify the costs of errors in pass/fail decisions. But is it possible to create defensible quantifications of such costs? Examiners will anyhow make exams of a certain length. Thus, at a certain point the examiner stops increasing the test length and implicitly accepts the corresponding error rate. Therefore, every finite exam contains an implicit assumption about how expensive it may be to avoid a decision error. It is worthwhile to make this assumption explicit and to investigate which information is needed for an evidence-based decision.

## The Probability of an Incorrect Fail

This section will use a classical test theory model to derive a formula that shows how the exam duration affects the expected number of students who fail incorrectly. Assume that the test can be lengthened or shortened without systematically altering the content type or difficulty, thus leaving the true scores the same. That is, the examiner would add or delete test items and change the duration of the examination proportionally. Lord and Novick (1968, Ch. 5) describe this as the model of homogeneous tests with continuous test length. Assume that the test, or a similar test, has been administered previously and that the duration of this administration was h hours. The grade on an exam with duration th>0 is written as $X_{t}=T+E_{t}$, where $X_{t}$ is the observed score, T is the true score, and $E_{t}$ is the measurement error. Assume that T and $E_{t}$ are independent normally distributed, with $T\sim N(\mu ,\tau^{2})$ and $E_{t}\sim N(0,\varepsilon_{t}^{2})$ and $X_{t}\sim N(\mu ,\sigma_{t}^{2})$. Following Lord and Novick, the reliability of the test will be defined as $\rho_{t}:=\tau^{2}/\sigma_{t}^{2}$. This is the classical test theory definition of the reliability coefficient, which is discussed in many psychometric textbooks (e.g., Gregory, 2007, p. 101; Lord & Novick, 1968, p. 61; Webb et al., 2006, formula 1.2b).

Assume that the examiner estimated from this previous administration the values of μ, $\sigma_{1}$, and $\rho_{1}$. If the duration of the exam is changed from duration 1h to duration th, then one would usually also change the number of test items proportionally. Assume that the error variance changes into $\varepsilon_{t}^{2}=\varepsilon_{1}^{2}/t$, which implies that the reliability of the test will change according to the Spearman–Brown formula (Lord & Novick, 1968, p. 112):

$$\rho_{t}=\frac{t\rho_{1}}{1+\left(t-1\right)\rho_{1}}$$

Assume that the cutoff for passing the test is some real number a. That is, a student fails for the exam if $X_{t}<a$ and passes for the test otherwise. Similarly, we can compare the true score with a. If T<a, then the student should fail according to the true score, and otherwise the student should pass. Therefore, the student fails incorrectly if $X_{t}<a$ while T≥a. Denote the cdf of the univariate standard normal distribution with Φ() and the cdf of the bivariate standard normal distribution as $\Phi_{2}(x,y,r)$, where the third argument is the correlation. Since $\sigma_{t}=\tau /\sqrt{\rho_{t}}$, the probability that a random student fails is

$$\Phi \left(\frac{a-\mu }{\sigma_{t}}\right)=\Phi \left(\frac{a-\mu }{\tau }\sqrt{\rho_{t}}\right)$$

The correlation between $X_{t}$ and T is $\sqrt{\rho_{t}}$, therefore the probability of an incorrect fail is

$$F_{t}=\Phi \left(\frac{a-\mu }{\tau }\sqrt{\rho_{t}}\right)-\Phi_{2}(\frac{a-\mu }{\tau }\sqrt{\rho_{t}},\frac{a-\mu }{\tau },\sqrt{\rho_{t}})$$

Note that $\tau =\sigma_{1}\sqrt{\rho_{1}}$, which can be estimated from the test administration with duration h. If N students take the exam, the expected number of students who fail incorrectly would be $NF_{t}$. Given the parameters μ, $\sigma_{1}$, and $\rho_{1}$ and the cutoff a, this number can easily be computed for many different values of the duration t.

## The Costs of Measurement and the Costs Measurement Error

This section will incorporate costs in the model. As explained in the introduction, the model will only consider the costs in terms student time. Suppose that the study load of a course is L hours. A student who fails the course will usually have to study again. Assume that the additional study time is proportional, but not necessarily equal, to the study load. Denote the corresponding *restudy fraction* as Q. In principle this is a number that can be estimated empirically by asking the students how much time they spent on learning for the resit, but the current reality is that this fraction is usually unknown. Therefore, we will study several values of Q, e.g. 0.33, 0.5, and 1. The costs of the measurement errors in the student population with N students would therefore be $\mathrm{NLQ}F_{t}$. At the same time, the measurement itself will also have costs, namely all students have to make the exam of duration ht. The time costs of this are Nht. Thus, the total time costs in the population are

$$C_{t}=\mathrm{NLQ}F_{t}+\mathrm{Nht}$$

To find the optimum duration, we have to minimize this in t. The solution does not depend on N, therefore we may simply use, for instance, N=100 everywhere. It is often convenient to analyze rather the normed costs $c_{t}:=C_{t}/\mathrm{Nh}$ and use the *costs quotient*S:=LQ/h, yielding

$$c_{t}=SF_{t}+t$$

If the restudy fraction Q is a random variable, independent of the observed scores and true scores, then its expectation E(Q) can be used in the same formula for $c_{t}$ with S:=LE(Q)/h.

## Example With a Real Exam

In a multiple-choice exam of one of my courses, the nominal study load was 112 hours, the cutoff was a=5.5, and the following estimates were found in an exam of approximately h=3 hours: μ=5.8, $\sigma_{1}=1.93$, and $\rho_{1}=0.80$. What would be the optimum exam length, assuming that these outcomes are representative for future exams? Furthermore, let us assume for simplicity here that the exam durations are limited to the integer numbers of 1, 2, 3, 4, or 5 hours (this was actually imposed by the management for logistic reasons). If we assume Q=1/3, we get the outcomes of Table 1. In this table, the minimum costs are obtained with an exam duration of 2 hours, shorter than the actual exam was. The reliability would then be 0.73, less than the usually defended standard 0.80. Both shorter and longer tests would cost the student population more time than this.

**Table 1.** Computation of Expected Number of Incorrectly Failed Students ($NF_{t}$), Costs of Measurement Errors ($\mathrm{NLQ}F_{t}$), Costs of Measurement (Nht) and Total Costs ($C_{t}$) in a Real Example, Assuming Q=1/3 and N=100.

Duration of exam in hours	t	$\rho_{t}$	$NF_{t}$	$\mathrm{NLQ}F_{t}$	Nht	$C_{t}$
5	1.67	0.87	6.0	225.2	500	725.2
4	1.33	0.84	6.7	250.1	400	650.1
3	1.00	0.80	7.6	285.4	300	585.4
2	0.67	0.73	9.1	341.3	200	541.3
1	0.33	0.57	12.1	450.9	100	550.9

If we consider Table 1 in more detail, we can see what happens. First, consider the reliabilities in column $\rho_{t}$. These are increasing with the test length, as they are computed with the Spearman–Brown formula. However, if we only require that the reliability is as high as possible, that is 1.0, this does not entail a practical criterion, as the Spearman–Brown formula would lead to a test of infinite length. Similarly, in the column $NF_{t}$ we see that the expected number of incorrectly failing students is decreasing if the test becomes longer. If we require that this number is minimal, that is 0, we end with an infinite test again. The same is true for the costs of measurement error, $\mathrm{NLQ}F_{t}$, which is proportional to $NF_{t}$. However, the measurement costs, Nht, have the opposite pattern; they increase with the test length, and their optimum would be a test with length 0. In the combined function $C_{t}$, these two opposite patterns lead to a function with a minimum at (in this case) 2 hours.

An obvious weakness in this computation is the unknown value of Q. Therefore, the computations were repeated with values 0.5, 0.75, and 1. With Q=0.5, the optimum would still be 2 hours, but with Q=0.75 it would be 3 hours and with Q=1.00 it would be 4 hours.

## Existence of Minimum Costs With Continuous *t*

**Figure 1.**
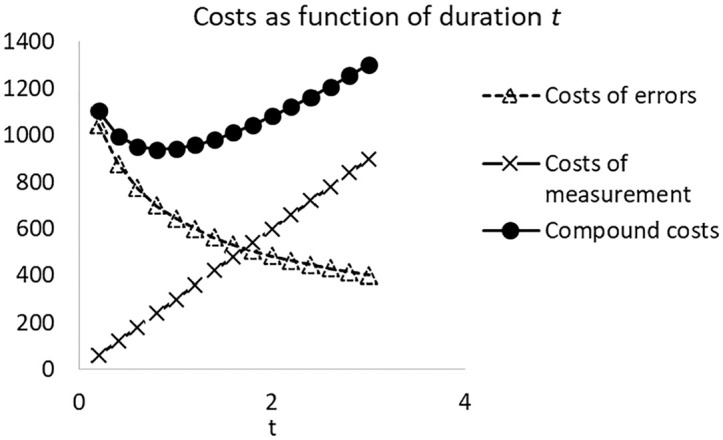
Costs of errors and costs of measurement as a function of the length of the test.

This section will show that for continuous t, the normed cost function $c_{t}$ has a minimum. The basic idea of the argument is illustrated in Figure 1. The costs of the errors are a decreasing convex function of the duration of the test, the costs of measurement are an increasing linear function of the duration of the test, and their sum is a U-shaped function. The will now be shown more formally.

Let us first establish that $F_{t}$ is a decreasing convex function of t. Note that

(1) $$\begin{array}{c} F_{t}=P\left(X_{t}<a,T\geq a\right)=\int_{a}^{\infty }P(X_{t}<a|T=s)f_{T}\left(s\right)\mathrm{ds}\\ =\int_{a}^{\infty }P(E_{t}<a-s|T=s)f_{T}\left(s\right)\mathrm{ds}\\ =\int_{a}^{\infty }P(E_{t}<a-s)f_{T}\left(s\right)\mathrm{ds}\\ =\int_{a}^{\infty }\Phi \left(\frac{a-s}{\varepsilon_{t}}\right)f_{T}\left(s\right)\mathrm{ds}\\ =\int_{a}^{\infty }\Phi \left(\frac{a-s}{\varepsilon_{1}}\sqrt{t}\right)f_{T}\left(s\right)\mathrm{ds} \end{array}$$

Now consider the partial derivative $\partial F_{t}/\partial t$. Since the function under the integral is continuously differentiable, we may interchange the order of differentiation and integration and differentiate under the integral. The derivative with respect to t of the first function inside the integral is

(2) $$\frac{\partial }{\partial t}\Phi \left(\frac{a-s}{\varepsilon_{1}}\sqrt{t}\right)=\varphi \left(\frac{a-s}{\varepsilon_{1}}\sqrt{t}\right)\left(\frac{a-s}{\varepsilon_{1}}\right)\left(\frac{1}{2\sqrt{t}}\right)$$

which is negative for s>a. Therefore, $\partial F_{t}/\partial t<0$, hence $F_{t}$ is decreasing in t.

The second derivative with respect to t is, if we write $b=(a-s)/\varepsilon_{1}$,

$$\frac{\partial^{2}}{\partial t^{2}}\Phi \left(\frac{a-s}{\varepsilon_{1}}\sqrt{t}\right)=-\varphi \left(b\sqrt{t}\right)\frac{\left(b^{3}t+b\right)}{4t^{3/2}}$$

For s>a we have b<0, which makes the second derivative positive. Therefore, $\partial^{2}F_{t}/\partial t^{2}>0$, hence $F_{t}$ is convex in t.

For the derivative of $c_{t}$ we have

$$\frac{\partial }{\partial t}c_{t}=S\frac{\partial }{\partial t}F_{t}+1$$

We can write the derivative in (2) as

$$\frac{b\varphi \left(b\sqrt{t}\right)}{2\sqrt{t}}$$

The limit of this for t→0 is −∞, and the limit for t→∞ is 0. Consequently,

$$\begin{array}{c} \underset{t\to 0}{\lim}\frac{\partial }{\partial t}c_{t}=-\infty \\ \underset{t\to \infty }{\lim}\frac{\partial }{\partial t}c_{t}=1 \end{array}$$

In sum, $c_{t}$ is a convex function for t>0, decreasing for values of t in a neighborhood of 0, and increasing for large value of t. Therefore, $c_{t}$ has a minimum in the positive real numbers.

Because $c_{t}$ is convex, it is easily minimized numerically. I have created the R package **exdur** with the function minexamcosts() that minimizes $c_{t}$ in t. It takes arguments S, $a,\mu ,\sigma_{1}$, and $\rho_{1}$. The outcomes of the minimization will be denoted as

$$\begin{array}{c} c_{\mathrm{opt}}:=\min\{c_{t}|t>0\}\\ t_{\mathrm{opt}}:=\underset{t>0}{\mathrm{argmin}}c_{t}\\ \rho_{\mathrm{opt}}:=\rho_{t_{\mathrm{opt}}} \end{array}$$

Thus, $\rho_{\mathrm{opt}}$ is the reliability of the test with duration that minimizes the costs. It will henceforth be called the *optimal reliability*. Another name could be *efficient* reliability, similar to Ellis (2013).

## Numerical Examples

For the case discussed earlier, with L=112, Q=1/3, h=3, a=5.5, μ=5.8, $\sigma_{1}=1.93$, and $\rho_{1}=0.80$, we obtain S=12.44, and the costs are minimized with $t_{\mathrm{opt}}=0.519$, yielding $\rho_{\mathrm{opt}}=0.675$ and $c_{\mathrm{opt}}=1.783$. Thus, the optimal exam duration would be about half the original duration, with a considerable lower reliability. The original duration was h=3 hours, so the optimal duration would be $ht_{\mathrm{opt}}=1.56$ hours.

**Figure 2.**
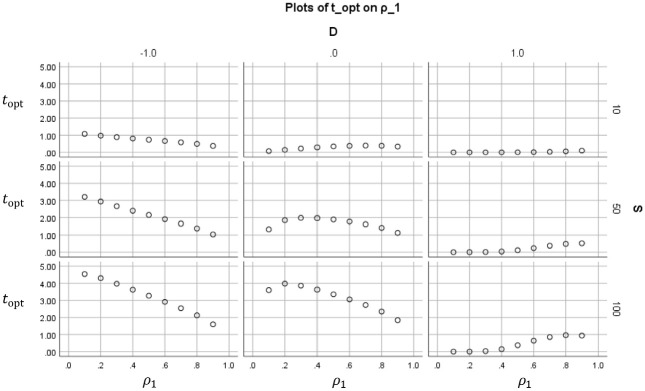
Optimal duration $t_{\mathrm{opt}}$ (vertical within subplots) that results if $c_{t}$ is minimized, plotted as a function of the original reliability $\rho_{1}$ (horizontal within subplots) The subplots are paneled by the ratio of cost S and the standardized cutoff D.

**Figure 3.**
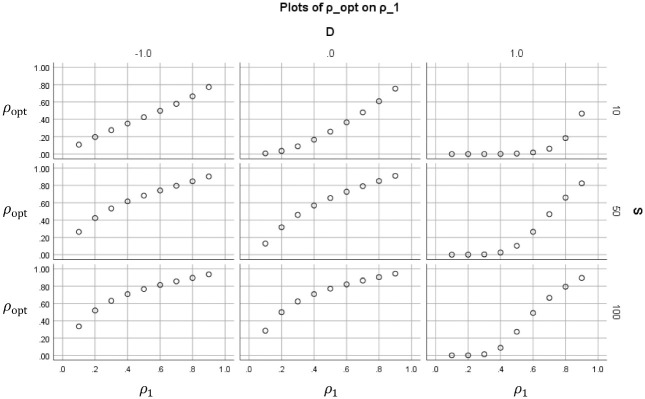
Optimal reliability $\rho_{\mathrm{opt}}$ (vertical within subplots) that results if $c_{t}$ is minimized, plotted as a function of the original reliability $\rho_{1}$ (horizontal within subplots). The subplots are paneled by the ratio of cost S and the standardized cutoff D.

**Figure 4.**
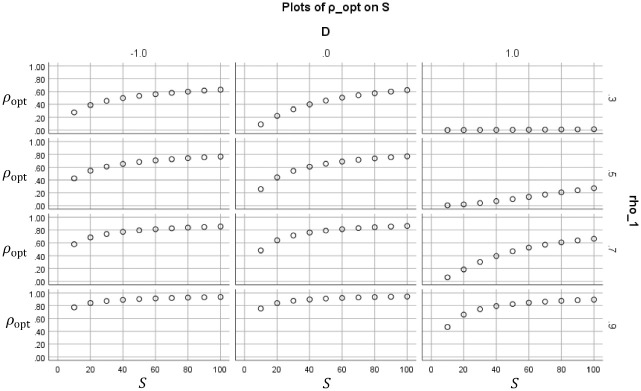
Optimal reliability $\rho_{\mathrm{opt}}$ (vertical within subplots) that results if $c_{t}$ is minimized, plotted as a function of the ratio of costs S (horizontal within subplots). The subplots are paneled by original reliability $\rho_{1}$and standardized cutoff D.

**Figure 5.**
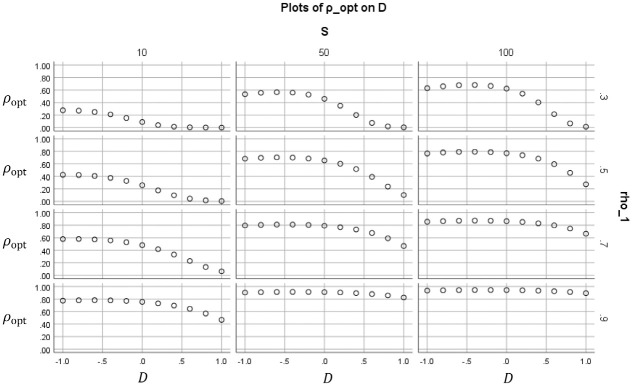
Optimal reliability $\rho_{\mathrm{opt}}$ (vertical within subplots) that results if $c_{t}$ is minimized, plotted as a function of the standardized cutoffD (horizontal within subplots). The subplots are paneled by original reliability $\rho_{1}$ and the ratio of costs S.

Figures 2 to 5 show how the value of the optimal reliability depends on S∈{10,50,100}, $\rho_{1}\in \{0.1,\ldots ,0.9\}$, and the standardized cutoff D:=(a−μ)/σ, D∈{−1,0,1}. The figures differ only in perspective and shown selection of values $S,\rho_{1},D$. In Figure 2, each subplot shows for a certain combination of (S,D) how the optimal duration depends on the original reliability $\rho_{1}$. These trends are decreasing if D=−1 (the mean is higher than the cutoff), peaked if D=0 (the mean is equal to the cutoff), and increasing if D=1 (the mean is lower than the cutoff). The optimum duration tends to be less than 1 (meaning that the test may be shortened) if the costs quotient is small (S=10) or students score low (D=1). In the other cases, usually $t_{\mathrm{opt}}>1$, which means that the test should be lengthened if one wants to minimize the costs. Figure 3 shows for the same cases the value of the optimal reliabilities $\rho_{\mathrm{opt}}$. Unlike $t_{\mathrm{opt}}$, these are increasing in $\rho_{1}$. Note that for low-cost quotient (S=10) the optimal reliabilities do not exceed .80 in these cases.

In Figure 4, each subplot shows for a certain combination of $\left(\rho_{1},D\right)$ how the optimal reliabilities $\rho_{\mathrm{opt}}$ depend on the costs quotient S. The optimal reliability increases with the costs quotient in each subplot, as one would expect. The same pattern holds for the optimal durations, but this is not displayed.

In Figure 5, each subplot shows for a certain combination of $\left(\rho_{1},S\right)$ how the optimal reliabilities $\rho_{\mathrm{opt}}$ depend on the standardized cutoff D. Roughly speaking, the optimal reliability decreases with the standardized cutoff, that is, it increases with the mean grade. However, this in not always true. For S≥50 and D≤−0.50 there is a trend in the opposite direction, with the optimal reliability slightly increasing. The same pattern holds for the optimal durations, but this is not displayed.

## Analysis of a Special Case

Consider the possibility that the mean of the grades is equal to the cutoff: μ=a. One might expect this approximately if students try to study just enough to pass the exam, a strategy described by Kooreman (2013) and Nijenkamp et al. (2016). This makes the cost function easier to study, and even more so since the costs have a simple expression: In this case we have

$$\begin{array}{c} F_{t}=\Phi \left(0\right)-\Phi_{2}\left(0,0,\sqrt{\rho_{t}}\right)\\ =\frac{\arccos\sqrt{\rho_{t}}}{2\pi } \end{array}$$

(Rose & Smith, 1996, in Weisstein, 2019). Thus

$$c_{t}=S\frac{\arccos\sqrt{\rho_{t}}}{2\pi }+t$$

If we write $u=\sqrt{\rho_{t}}$ and try to solve $\frac{\partial }{\partial u}c_{t}=0$, we get

$$\frac{S}{4\pi }\frac{\rho_{1}}{\left(1-\rho_{1}\right)}=\frac{u\sqrt{1-u^{2}}}{{\left(u^{2}-1\right)}^{2}}$$

**Figure 6.**
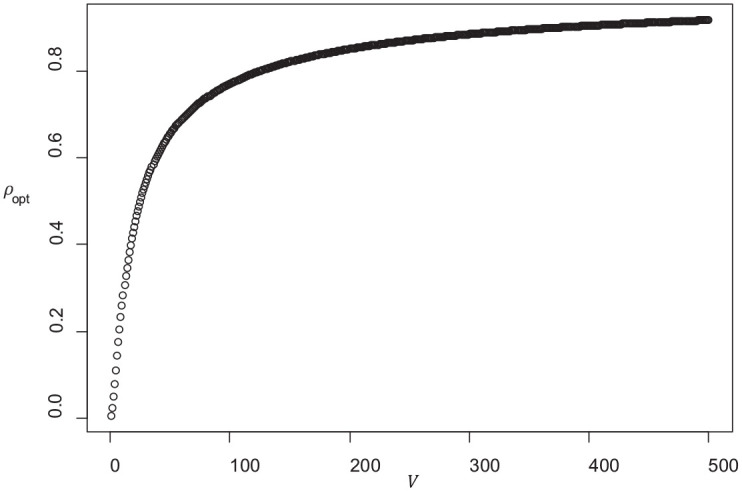
Reliability $\rho_{\mathrm{opt}}$ that results if $c_{t}$ is minimized, plotted as a function of $V=S\rho_{1}/\left(1-\rho_{1}\right)$.

Although I do not know an analytical solution in u, the function at the right side is increasing, and therefore the value of $\rho_{\mathrm{opt}}$ at which the costs are minimized is an increasing function of $S\rho_{1}/\left(1-\rho_{1}\right)$. Figure 6 shows the optimal reliability $\rho_{\mathrm{opt}}$ as a function of $V:=S\rho_{1}/\left(1-\rho_{1}\right)$. For integer values of V between 1 and 380, an approximation of $\rho_{\mathrm{opt}}$ with absolute deviation less than 0.01 can be obtained with the rational function

$$\rho_{\mathrm{opt}}\approx \frac{0.000234873\;*\;V^{3}+0.039478353\;*\;V^{4}}{1+3.0640\;*\;V+2.697293\;*\;V^{2}+0.831824\;*\;V^{3}+0.042123\;*\;V^{4}}$$

Because of the simplicity of this case, it can be used a default model if one wants to avoid arbitrary assumptions on μ and σ. That is, it may be viewed as a kind of “middle-of-the-road” model for cost efficient examination, even if the mean grade is not exactly known. In the numerical examples of the previous section, the optimal reliabilities obtained with this simplified model correlated 0.995 with the optimal reliabilities obtained from a double sided version of the costs function, where the probability of incorrectly failing the exam is replaced with the mean probability of incorrectly passing the exam and an incorrectly failing the exam. Thus, the simplified formula can also be viewed as a version that is more balanced with respect to the costs of errors.

In the exam of the earlier example, we had L=112, h=3, $\rho_{1}=0.80$, and we assumed Q=1/3. Minimization of $c_{t}$ with μ=a results in $\rho_{\mathrm{opt}}=0.6534$ and $c_{\mathrm{opt}}=1.718$. If we apply the approximation above, we get V=49.8 and $\rho_{\mathrm{opt}}\approx 0.6586$. This is close to the outcomes that were previously obtained with μ=5.6,a=5.5, where we found $\rho_{\mathrm{opt}}=0.675$.

## A Model With Score-Dependent Costs

As pointed out by a reviewer, it is possible that the amount of time that a student uses to prepare for the resit depends on the student’s score on the first attempt. This will now be modelled. Assume that, for students who fail the exam, the restudy fraction Q is a random variable, and that its conditional expectation is proportional to the difference of the student’s score and the cutoff:

$$E\left(Q|X_{t}\right)=\beta (a-X_{t})$$

if $X_{t}<a$. The expected costs per student, including the extra study time of students who failed incorrectly and the time costs of the exam, normed by assuming h=1, are

$$c_{t}=L\;*\;E\left(Q|X_{t}<a,T>a\right)F_{t}+t$$

For a student who incorrectly fails the exam, the expected restudy fraction would be

(3) $$\begin{array}{c} E\left(Q|X_{t}<a,T>a\right)=E\left(\beta (a-X_{t})|X_{t}<a,T>a\right)=\\ \beta a-\beta \;*\;E\left(X_{t}|X_{t}<a,T>a\right)=\\ \beta a-\beta \;*\;E\left(E\left(X_{t}|X_{t}<a,T\right)|T>a\right) \end{array}$$

The conditional distribution of $X_{t}|T$ is $N(T,\varepsilon_{t}^{2})$, and using the fact that the expectation of a truncated normal can be expressed with the inverse Mill’s ratio, we get

$$E\left(X_{t}|X_{t}<a,T\right)=T-\varepsilon_{t}\frac{\varphi (\frac{a-T}{\varepsilon_{t}})}{\Phi (\frac{a-T}{\varepsilon_{t}})}$$

and the expected grade of students who failed incorrectly is

$$E\left(E\left(X_{t}|X_{t}<a,T\right)|T>a\right)=E\left(T-\varepsilon_{t}\frac{\varphi (\frac{a-T}{\varepsilon_{t}})}{\Phi (\frac{a-T}{\varepsilon_{t}})}|T>a\right)=$$

$$E\left(T|T>a\right)-E\left(\varepsilon_{t}\frac{\varphi (\frac{a-T}{\varepsilon_{t}})}{\Phi (\frac{a-T}{\varepsilon_{t}})}|T>a\right)=$$

$$\mu +\tau \frac{\varphi \left(\frac{a-\mu }{\tau }\right)}{1-\Phi \left(\frac{a-\mu }{\tau }\right)}-E\left(\frac{\varepsilon_{1}}{\sqrt{t}}\frac{\varphi (\frac{a-T}{\varepsilon_{1}}\sqrt{t})}{\Phi (\frac{a-T}{\varepsilon_{1}}\sqrt{t})}|T>a\right)$$

Here, the first two terms do not depend on t. With $x:=\left(a-T\right)\sqrt{t}/\varepsilon_{1}$ and G(x):=φ(x)/Φ(x) (the inverse Mill’s ratio), the variable inside the conditional expectation operator is (a−T)G(x)/x, with x<0. Now G(x)/x is decreasing, which follows from the fact that the derivative of G(x)/x is $-x^{-2}G\left(x\right)-G\left(x\right)-x^{-1}G(x{)}^{2}$, which is negative for x<0 if $-(1+x^{2})/x\geq G(x)$. The latter inequality was proved true by Gordon (1941, p. 365). Consequently, the component $E\left(Q|X_{t}<a,T>a\right)$ of $c_{t}$ is decreasing in t. It was already established that $F_{t}$ is decreasing. Thus, the expected costs of errors are decreasing with the exam duration t, as one would intuitively expect. Gordon also showed that $\underset{x\to \infty }{\lim}x/G(-x)=1$, which implies that (3), as a function of t, has a right asymptote. Therefore the part $E\left(Q|X_{t}<a,T>a\right)F_{t}$ of $c_{t}$ must be decreasing with a right asymptote.

**Figure 7.**
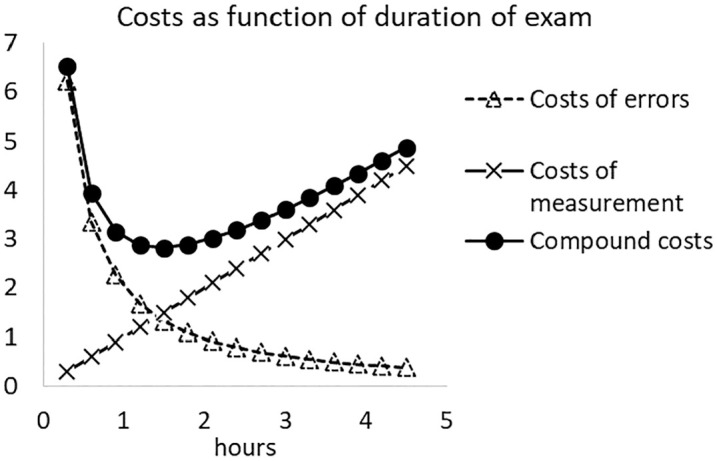
Costs of errors and costs of measurement as a function of the exam duration in a case of score-dependent costs.

As a real data example, consider the case discussed earlier with L=112, h=3a=5.5, μ=5.8, $\sigma_{1}=1.93$, and $\rho_{1}=0.80$. Assume furthermore that a student with $X_{t}=0$ will study the course as if it is a new course: $E\left(Q|X_{t}=0\right)=1$, which implies $\beta =a^{-1}$. The expected costs of errors, costs of measurements, and compound costs $c_{t}$ are plotted as a function of the duration of the exam (ht hours) in Figure 7. The compound curve has again a minimum, and numerical minimization of the compound costs yields $t_{\mathrm{opt}}=0.49$, which corresponds to an exam duration of 1.5 hours. This is approximately equal to the outcome of the first model.

## Sensitivity to Sampling Error

The numerical example was based on empirical estimates of μ, $\sigma_{1}$, and $\rho_{1}$ in one exam. How sensitive is $t_{\mathrm{opt}}$ to sampling error? The data were based on 190 students. A simulation was performed in which 1,000 bootstrap samples of 100 subjects (much less than the real sample) were drawn with replacement from this data set. For each sample, the sample mean and same standard deviation of the grades was computed, and $\rho_{1}$ was estimated as Cronbach’s alpha. Next, $t_{\mathrm{opt}}$ was computed for each sample, using L=112 and fixed Q=1/3 and h=3. The percentile-based 90% confidence intervals for μ, $\sigma_{1}$, $\rho_{1}$, and $t_{\mathrm{opt}}$ were [0.78, 0.86], [5.4, 6.09], [1.73, 2.19], and [0.44, 0.56], respectively. The distribution of $t_{\mathrm{opt}}$ was slightly skewed with more high outliers than low outliers. The minimum was 0.39 and the maximum was 0.66. The mean and median of $t_{\mathrm{opt}}$ were both equal to 0.50, and the standard deviation was 0.04. The confidence interval of $t_{\mathrm{opt}}$ corresponds to an exam duration of 1.31 to 1.67 hours. Thus, in this example, where the test had parameters such that the costs are minimized with a duration of 1.56 hour, if random samples of 100 subjects are drawn, the variability in sample means, sample standard deviations, and sample reliability coefficients produces estimates for the optimal duration that vary between 1.31 and 1.67 hours in 90% of the samples.

## Discussion

What is the best duration for an exam? How reliable should exam scores be? How is this affected by the study load of the course? In the construction of exams and tests it is often emphasized that they should be reliable. If this were the only criterion, exams should be very long, and in theory even infinitely long. The practical decision whether a certain test is long enough is inevitably partially based on intuitive reasoning and personal experience, since there are hitherto no formal models that justify a less than perfect reliability. This article shows that such models are possible and entail realistic outcomes.

The model created here assumes a classical test theory model, with normally distributed true scores and error scores. From the mean, standard deviation, and reliability of the test, we can compute the probability of erroneous fail on the exam. To quantify the costs of such errors, it was assumed that the students who fail will lose a fraction of the nominal study load because they have to study for the resit. These hours were called the costs of errors. On the other hand, a long test will also consume time from students, and this was called cost of measurement. The compound costs were now defined as the costs of errors plus the costs of measurement. The compound costs were expressed as a function of the test length, and its minimum is easily obtained numerically.

The models make clear that there are many factors contributing to the costs of the exam. If a course is repeated yearly, the examiner can presumably have a reasonable estimate of the values of $\rho_{1},\mu$, and σ that can be expected on the next exam. However, the restudy factor Q is probably unknown. It would be interesting to have empirical research estimating the distribution of this factor, which would facilitate more substantiated costs computations in practice. Even without this, we can have some intuitive arguments. For example, in some exams the time between the date on which the grades are published and the date of the resit is simply not enough to get Q=1 within regular working hours. In some other exams the time is half a year, which makes a high value of Q more likely.

What if there is no resit? In that case the model cannot be applied literally. However, one could argue that in this case a student who fails loses the time already spent in the course, which is probably proportional to the study load. Therefore, the same mathematical model would still be appropriate, albeit with a different interpretation of Q. Indeed, the model can also be applied with other definitions of costs if these are proportional to the study load and the exam duration, such as emotional stress.

A limitation of the two models discussed is that students who incorrectly pass the exam ($X_{t}>a,T<a$) were not counted in the costs. A reviewer argued that this could bias results in favor of incorrect passes. The reason for the omission was that the time costs of this event are less clear. One solution is to compute the costs in the first model with the assumption that the mean of the grades is equal to the cutoff: μ=a. In that case both error events $V_{t}:=[X_{t}<a,T>a]$ and $W_{t}:=[X_{t}>a,T<a]$ have the same probability. It was shown that this leads to the cost function $c_{t}=S(2\pi {)}^{-1}\arccos\sqrt{\rho_{t}}+t$. A second solution would be to include the probability of incorrect passes $H_{t}:=P\left(X_{t}>a,T<a\right),$ and give it the same weight as incorrect fails. Applied to the first model, this leads to the cost function $c_{t}=S(F_{t}+H_{t})+t$. Obviously, if μ≈a this leads to similar outcomes as $c_{t}=2SF_{t}+t$, which is equivalent to doubling the study load L in the original model. A third solution would be to include $H_{t}$ with a separate weight for incorrect passes. For example, one could argue that a student who passes while T<a misses knowledge of measure a−T. The worth of this would be proportional to γL(a−T) for some parameter γ. For example, since the student would pass with T=a, which apparently was worth study load L, one might set the worth of the missing knowledge to L(a−T)/a, which corresponds to $\gamma =a^{-1}$. The costs of these errors would therefore be $L\gamma E(a-T|X_{t}>a,T<a)H_{t}$. This has a similar structure as the error costs of the second model. An overview of the various cost functions is given in Table 2. This article focussed on cost functions numbered 1a, 1a(i), 2a, and 2a(ii) in the table, but the other cost functions can be treated similarly.

**Table 2.** Overview of Cost Functions.

Function	(a) Incorrect fails	(b) Incorrect passes	(c) Both
1	$L\alpha F_{t}+t$	$L\alpha H_{t}+t$	$L\alpha (F_{t}+H_{t})+t$
2	$L\beta E\left(a-X_{t}|V_{t}\right)F_{t}+t$	$L\gamma E(a-T|W_{t})H_{t}+t$	$L\beta E\left(a-X_{t}|V_{t}\right)F_{t}+$ $L\gamma E(a-T|W_{t})H_{t}+t$

*Note*. L is the study load, Q is the restudy fraction, a is the cutoff, $X_{t}$ is the observed test score, T is the true score, t is the exam duration, $V_{t}:=[X_{t}<a,T>a]$, $W_{t}:=[X_{t}>a,T<a]$, $F_{t}:=P\left(V_{t}\right)$, $H_{t}:=P\left(W_{t}\right)$. The parameters α,β,γ can be set a priori or estimated from the distribution of Q in data, e.g. α:=E(Q). Special cases can be created by setting (i) a=μ, (ii) $\beta =a^{-1}$, and (iii) $\gamma =a^{-1}$.

Another limitation of this study is the usage of the Spearman–Brown formula to predict the reliability under changing test length. It is known that this formula holds if the test items are parallel, but in many cases test items are not parallel. The Spearman–Brown formula holds actually more generally if the items are “essentially parallel” (i.e., essentially tau-eq24 with equal variances), but even this condition is rarely satisfied. The Spearman–Brown formula has also been derived by Raju and Oshima (2005) within an item response theory framework under the assumption that the average item information function does not change if items are added, which is again quite restrictive. However, the Spearman–Brown formula can also be applied to subtests instead of items, where each subtest consists, for example, of 10 items. The assumption of subtests that are approximately essentially parallel is not necessarily unrealistic. The important restriction here is that the items used in long versions of the test should be similar in kind to the items used in short versions of the test. A situation in which this is realistic is if the items are drawn randomly from a large pool of items, particularly if this pool is known to be unidimensional according to an item response theory model. To support this with an example, a simulation was conducted in which items were drawn from a pool of 1,000 items that satisfies the 2-parameter logistic model with discrimination parameters uniformly distributed between 0.5 and 2.5 and difficulty parameters uniformly distributed between −1.5 and 1.5, and these items were used to generate random tests with lengths between 10 and 50 items, in steps of 5 items. For each of these test lengths, 1,000 tests were generated with items randomly drawn from the pool, and the reliability was computed for each test by numerical integration, assuming a standard normal distribution of the latent ability. For test lengths 10, 15, 20, 25, 30, 35, 40, 45, and 50, the average reliabilities were .7417, .8106, .8526, .8775, .8955, .9096, .9199, .9282, and .9349, respectively. These values fit the Spearman–Brown formula almost perfectly: if any of these average reliabilities is predicted from any other one, the error is at most 0.0025. Thus, even though the individual tests might not satisfy the Spearman–Brown formula, the *expected* reliability across random test versions can still be predicted very well with the Spearman–Brown formula in this example. Consequently, the arguments of the previous sections can be applied here too. It is beyond the scope of this article to determine under which conditions this generalization of the Spearman–Brown formula is appropriate, but a simulation like this can be conducted easily once the item parameters are known.

The Spearman–Brown formula does not account for possible practice and fatigue effects that might influence the reliability if the test length is changed. I do not know of a model that describes the effect of this on the item parameters, but if these effects were strong then this would also invalidate the increasingly popular methods of computerized adaptive testing (e.g., Van der Linden & Glas, 2000), which invariably assume that the ability and item parameters are not affected by the test length.

Thus, for the first time we have now a mathematical tool that allows us to estimate the optimal length of an exam from data of earlier similar exams. The tool is simple and easy to use, but obviously there are some limitations:

- The computation requires knowledge of the distribution of Q, the fraction of the nominal study load that the student will spend on preparing for the resit, and its regression on observed and true scores. For a realistic application of the model, it is desirable to study this empirically in future research.
- The computation ignored many other costs, such as extra financial costs if failing leads to dropping out of the program, the reputation damage when students with low true scores pass, emotional stress, costs of the school to make longer exams, and so on.
- The distribution of item characteristics should remain the same if the test is lengthened or shortened, such that the Spearman–Brown formula is valid. To avoid floor and ceiling effects, it may be better to work with an item response theory model instead of classical test theory.

More detailed and realistic computations may be possible if all these components are added to the model, and the important conclusion from this article is that there is proof of concept.
